# One out of four recruits drops out from elite military training due to musculoskeletal injuries in the Netherlands Armed Forces

**DOI:** 10.1136/bmjmilitary-2020-001420

**Published:** 2020-03-05

**Authors:** Iris Dijksma, WO Zimmermann, E-J Hertenberg, C Lucas, MM Stuiver

**Affiliations:** 1 Primary Health Care, Defense Health Care Organisation, Netherlands Ministry of Defense, Utrecht, Utrecht, The Netherlands; 2 Master Evidence Based Practice in Health Care, Clinical Epidemiology, Biostatistics and Bioinformatics, Amsterdam UMC - Location AMC, Amsterdam, North Holland, The Netherlands; 3 Department of Sports Medicine, Royal Netherlands Army, Netherlands Ministry of Defense, Utrecht, Utrecht, The Netherlands

**Keywords:** epidemiology, primary care, rehabilitation medicine, sports medicine

## Abstract

**Introduction:**

Musculoskeletal injuries (MSIs) are among the main causes of dropout from military training. The main purpose of this study was to provide an overview of dropout rates and MSI incidence rates during elite military training. Second, this study aimed to explore restricted training days due to MSIs and to describe MSI-care by military physicians.

**Methods:**

In a retrospective observational study, we collected dropout rates and injury surveillance data from the electronic patient records of two elite units of the Netherlands Armed Forces (NAF): the Royal Netherlands Marine Corps (RNLMC) and the Airmobile Brigade (AMB), from 1 January 2015 until 31 December 2017.

**Results:**

In the RNLMC, total dropout rate was 53.9% and dropout due to MSIs was 23%. The most frequently affected locations were foot, knee and leg. In the AMB total dropout rate was 52.6% and dropout due to MSIs was 25%. In the AMB, the most frequently affected locations were back, knee and leg. Average restricted training days due to MSIs ranged between 8.3 and 20.8 days/injury. MSI-care by military physicians consisted mostly of the provision of injury-specific information and (self-)management options, imposing a specific activity restriction and referral to physiotherapy.

**Conclusion:**

Our study findings showed that one out of four recruits who dropout from elite military training in the NAF, do so due to MSIs. Redesigning training programmes with the objective to reduce MSIs should be given high priority, as this may reduce dropout substantially.

**Key messages**

- The findings of this study can serve as the baseline information required to design and evaluate injury prevention programmes.
- This study provides an overview of dropout rates and musculoskeletal injury incidence rates during elite basic military training.
- This study explored restricted training days due to musculoskeletal injuries and summarises the use of imaging and primary care interventions by military physicians.
- This study presents baseline data from which the effectiveness of future interventions may be assessed.

## Introduction

Despite extensive pre-enlistment psychological and physical fitness assessments to select eligible recruits, ample provision of information and training protocols designed by experts, attrition rates of elite basic military training courses in the Netherlands Armed Forces (NAF) remain substantial.^1^ Globally, musculoskeletal injuries (MSIs) are among the main causes of dropout from military training.^2 3^

In a previous study including 22% of all active duty personnel of the total NAF, we found that MSIs of the back (6.73 per 100 person-years (py), 95% Confidence Interval (CI): 6.39 to 7.10), knee (5.04 per 100 py, 95% CI: 4.74 to 5.35) and foot (4.79 per 100 py, 95% CI: 4.50 to 5.10) were the most reported injuries in 2014–2016. Furthermore, injuries of the knee and back accounted for the most limited duty days and thus lost productivity costs in this population. Impact in terms of lost productivity costs due to MSIs in versed personnel were estimated to amount to at least € 1.1 million per year.^4^

The initial military training of the Royal Netherlands Army Airmobile Brigade (AMB) and Marine recruit training of the Royal Netherlands Marine Corps (RNLMC) are mentally and physically highly demanding courses that span approximately half a year. In this population of recruits, the incidence of MSIs—and corresponding impact—is likely to be even higher than in versed personnel.^5^ Spikes in load and a rapid increase in chronic workload can lead to symptoms such as soft tissue injuries, bone stress injuries and joint sprains.^6^ Spikes in load during military training are likely more problematic when baseline fitness of recruits entering military training is low. In fact, low baseline fitness of recruits entering basic military training has been shown to be associated with an increased risk of MSIs and attrition from military training.^7 8^ The *Translating Research into Injury Prevention Practice* framework states that injury surveillance is the crucial first step towards injury prevention.^9^ Until this date, epidemiological data on MSIs in military trainees in the NAF have not been published.

Once an MSI occurs, evidence-based and personalised primary care is fundamental to decrease the risk of dropout due to the MSI. Military physicians have a preeminent role in this process as they are the first contact possibility and gatekeepers of healthcare, deciding whether, for example, a referral to the physiotherapy department is required. Military physicians provide care according to the guidelines of the Dutch College of General Practitioners.^10^ In civilian care, common pitfalls in MSI-care include: overuse of imaging, overuse of surgery, overuse of opioids and failure to provide injury-specific information and management options.^11^ It is unknown to what extent these pitfalls also apply to the defence healthcare organisation when treating military trainees with MSIs.

The main purpose of this study was to provide an overview of dropout rates and MSI incidence rates during elite basic military training of two elite units. Secondary, this study aimed to explore restricted training days due to MSIs and to summarise the use of imaging and primary care interventions by military physicians.

## Methods

In this retrospective observational study, data on person-time in training, passing rates and MSI occurrence were collected and combined from consecutive elite military training cohorts of the RNLMC and AMB from 1 January 2015 until 31 December 2017. The department of human resources provided data on person-time in training, including reasons for dropout. We defined person-time in training as the total number of days during which individuals were in military training, within the time frame of this study. Sometimes recruits drop out from a particular training course and resume training in another cohort; these so-called doubles were described as frequencies and proportions.

For a sample description at baseline, physical training instructors provided the results of the standard military physical fitness tests performed at the start of the elite military training courses. These data included maximal aerobic speed (metres/seconds, measured by a 12 min Coopertest), maximal number of push-ups in 2 min, and maximal number of sit-ups in 2 min. Additionally, an independent medical assistant extracted medical encounter data from the electronic patient records for the period in which the recruits were in military training, according to clearly outlined instructions. This included information regarding incident cases of MSIs, date of diagnosis, acute or gradual onset, number of restricted training days and a description of the interventions provided by the primary care military physicians. The assistant was blinded to the training outcome. Incident MSI cases were defined as an injury in any body part diagnosed with an *International Classification of Primary Care-code (ICPC-2) L-code ‘musculoskeletal injury’*, as registered by a military physician in the electronic patient records within the training period.^12^ Individuals stayed at risk for subsequent MSIs and continued to contribute person-time for another MSI once an MSI was diagnosed, as different MSIs can occur within an individual over time. Repeated records of an MSI within a time span of ≤3 months were considered to be follow-up consultations, rather than new cases, and therefore not included as incoming analysis. Acute injuries (defined as those caused by a single abrupt overload of the tissue or joint with sudden onset and a known cause) and gradual onset injuries (defined as those resulting from long-term energy exchanges resulting in cumulative micro-trauma over time) were extracted as interpreted and registered by a military physician.^13^

We restricted the exploration of MSI-care to the first injury as treatment protocols for second or recurrent injuries might be influenced by the applied treatment for the first injury and its outcome. MSI-care by military physicians was classified into eight categories, which were determined a priori: medication (eg, short-term pain reduction medication: acetaminophen, non-steroidal anti-inflammatory drug (NSAID)), specific activity restriction (eg, 5 days off from loaded marches), mobility exercises, wait-and-see policy, referral to physiotherapy, referral for imaging (eg, X-ray), provision of injury-specific information and (self-)management options and a referral for customised insoles. When a military physician prescribed the duration of a specific activity restriction in general terms, we assigned it an a priori defined duration that, in our clinical experience, corresponds best to the general recommendation. Thus, for a restriction of ‘several weeks’, we recorded 21 days in the database, and for ‘several months’, we recorded 91 days.

Data aggregation was done by the first author (ID) and checked by a military medical doctor (EJH). We explored the prescribed primary care interventions for back injuries, lower- and upper extremities per military unit separately.

The Medical Research Ethical Committee (MREC) of the University of Utrecht, The Netherlands, waived this study from formal medical ethical approval (protocol number: 17–631/C). Data were pseudonymised prior to analysis.

### Statistical methods

For statistical analysis, R V.3.5.3 was used in RStudio.^14^ Sample characteristics were summarised as frequencies and percentages for nominal variables, mean and SD for continuous variables with an approximate normal distribution and median and IQRs for continuous variables with a skewed distribution and ordinal variables. Dropout rates and dropout due to MSIs were described as percentages of the total number of persons in training. Acute and gradual onset injuries were described as a percentage of all MSIs. We calculated incidence density rates per 100 person-years (100 py), with a corresponding 95% CI.^15^ MSI incidence density rates are presented per body region for the RNLMC and AMB separately. As ‘shin pain’ is part of the code for leg/thigh complaints but is not covered by a unique ICPC-2 code, one researcher (ID) ran a query on the following terms in the diagnosis column: shin*, medial tibial stress syndrome (MTSS), MTSS, chronic exertional compartment syndrome (CECS), CECS, lower leg*. The retrieved records were interpreted and classified as shin pain or other leg/thigh complaints by the same researcher. We explored the number of training days with activity restrictions due to the MSIs with the highest incidence density rates and presented these as the average number of days per injury. When a military physician-prescribed activity restrictions of ‘several weeks’ we computed 21 days, and in case of prescribing ‘several months’, we computed 91 days. Lastly, primary care treatments are summarised descriptively and evaluated on common pitfalls; overuse of imaging, overuse of surgery, overuse of opioids, and failure to provide injury-specific information and management options.

## Results

### Royal Netherlands Marine Corps

Our sample included 482 recruits of the RNLMC recruit training. The total dropout rate was 53.9%, dropout due to MSIs was 23%, and 68% of all recruits suffered from one or more MSIs during the training period. Twenty-four per cent of the MSIs were acute and 48% had a gradual onset. The most frequently affected locations were foot, knee and leg. Forty-seven per cent led to specific activity restrictions and average restricted training days for the top three locations were: 20.8, 10.1 and 9.67 days/injury for foot, knee and leg, respectively. The most applied treatment strategies were specific activity restriction (34.6%), referral to physiotherapy (21.9%), medication (20.0%), provision of injury-specific information and (self-)management options (8.3%), and wait-and-see policy (6.3%).

### Airmobile Brigade

Our sample included 703 recruits of the AMB recruit training. The total dropout rate was 52.6%, dropout due to MSIs was 25%, and 44% of all recruits suffered from one or more MSIs during the training period. Thirty-two per cent of the MSIs were acute and 51% had a gradual onset. The most frequently affected locations were back, knee and leg. Thirty-seven per cent led to specific activity restrictions and average restricted training days for the top three locations were: 11.6, 8.3 and 20.9 days/injury, for back, knee and leg, respectively. The most applied treatment strategies were the provision of specific injury information and (self)-)management options (32.7%), specific activity restriction (20.1%), referral to physiotherapy (14.5%), medication (11.9%) and wait-and-see policy (6.1%).

The sample baseline characteristics of both military units are listed in Table 1. MSI incidence density rates per body region per 100 py are presented in Table 2. Through close inspection of the data, we identified five cases as follow-up consultations that we excluded from the incidence rate analysis. In both military units, referrals for imaging were scarce (3.9%) and mostly provided for injuries in lower extremities (eg, suspected stress fractures of the foot, ankle sprains). In general, the most frequent combination of treatments contained a specific activity restriction combined with referral to physiotherapy. Acetaminophen and NSAID were exclusively prescribed as short-term pain medication, whereas opiates were not.

**Table 1:** Sample baseline description

	Royal Netherlands Marine Corps recruit training (*n*=482)	Airmobile Brigade recruit training (*n*=703)
Male	100%	99.7%
Doubles, n (%)	64 (13%)	80 (11%)
Age, mean (SD)	20.6 (2.34)	20.7 (2.70)
	n=*99*	n=*328*
Maximal aerobic speed, mean (SD)	3.97 (0.16)	3.94 (0.24)
Push ups in 2 min, med (IQR)	57 (50–57)	48 (41–55)
Sit ups in 2 min, med (IQR)	56 (52–61)	54 (48–59)

n, number; med, median.

**Table 2:** Musculoskeletal injury incidence rates per 100 person-years, per body region

Incidence rate per 100 person-years (95% Confidence Interval)		
	Royal Netherlands Marine Corps recruit training (*n*=482)	Airmobile Brigade recruit training (*n*=703)
Ankle	26.0 (19.2 to 35.3)	16.8 (12.2 to 23.4)
Arm, elbow, wrist	7.61 (4.32 to 13.4)	4.68 (2.52 to 8.70)
Back	22.2 (15.9 to 30.9)	29.9 (23.4 to 38.3)
Foot	64.7 (53.3 to 78.6)	22.9 (17.3 to 30.3)
Hand	12.7 (8.19 to 19.7)	5.15 (2.85 to 9.29)
Hip	5.08 (2.54 to 10.2)	2.34 (0.97 to 5.62)
Knee	62.2 (51.0 to 75.8)	28.1 (21.8 to 36.2)
Leg/thigh	46.3 (36.8 to 58.3)	28.1 (21.8 to 36.2)
Neck	1.90 (0.61 to 5.90)	2.34 (0.97 to 5.62)
Non-specified region	32.4 (24.5 to 42.6)	28.5 (22.2 to 36.7)
Shin pain	43.8 (34.6 to 55.4)	18.7 (13.7 to 25.5)
Shoulder	9.52 (5.74 to 15.8)	6.55 (3.88 to 11.1)

## Discussion

This study aimed to provide an overview of dropout rates and MSI incidence rates during elite basic military training of two elite units of the NAF to explore restricted training days due to MSIs and to summarise used primary care interventions for those MSIs. Our study findings showed that one out of four recruits drops out of elite military training due to MSIs. Injuries of the foot, knee, leg and back formed the majority and mostly had a gradual onset. MSI-care predominantly consisted of the provision of injury-specific information and (self-)management options, imposing a specific activity restriction and referral to physiotherapy.

Notably, we observed higher MSI incidence density rates in the RNLMC compared with the AMB, which cannot be explained by baseline fitness, and are presumably caused by training specifics and the interplay between workload, personality characteristics and individual preparation in the pretraining phase.^16 17^ Although clarifying these differences was beyond our study scope, we can speculate about underlying mechanics to explain this finding. For example, the incidence rate of foot complaints was almost threefold higher in the RNLMC than in the AMB. We expect that this finding can be explained partly by the fact that Marine recruits frequently suffer from dermatological complaints of the foot such as pitted keratolysis. Pitted keratolysis is a bacterial skin infection of the foot, caused by excessive sweating and use of occlusive footwear. The infection is fairly common, especially when wet shoes, after ditch marches and other amphibious exercises, are worn for extended periods.^18^ Further, we speculate that higher acute workloads in the RNLMC (greater distances with heavier load) and/or timely access to military healthcare (exclusively marine training centre base) and/or group culture (eg, recruits are encouraged to visit the physician) may contribute to this finding. Prospective cohort studies are needed to further investigate and explain the difference in MSI incidence rates between the two military units.

As expected, MSI incidence rates in military trainees were much higher—roughly a 10-fold—than MSI incidence rates in active duty personnel of the NAF.^4^ The majority of MSIs in military trainees were injuries of the lower extremity and the back, with a gradual onset, which can be considered training-related injuries. Both in sports and in the military, preventing *training load error* is considered an injury prevention strategy with a high priority.^2 9 19 20^ Military athletes with well-developed physical capabilities are less likely to develop MSIs during increasing workload, compared with recruits with low physical fitness and capabilities.^21^ At the same time, maximal adaptation to the training load will be achieved only when there is an optimal balance between load-capacity and load. General but essential factors that should therefore—at least—be consciously considered in programming training and workload are recruits’ baseline physical fitness, the available time and means, and the level of fitness that is required at the end of the training period.^6^ Those factors can be manipulated by designing and offering physical preparation protocols, adapting training planning and focus to individual recruits or subgroups of recruits with comparable capacity, and by using a flexible rather than a fixed training period. Implementing this approach will require time and effort, but is likely to increase the number of recruits completing military training successfully.^8^

When we compare our observations concerning MSI-care to the recommendations for primary care and common pitfalls as proposed by Lin *et al*, we found no indications for overuse of imaging, opioids or failure to provide injury-specific information and management options. Thus, on an aggregated level, the common pitfalls in MSI-care did not apply in our study.^11^ Regarding dropout from training but also dropout from training in particular due to MSIs, treatment of those MSIs is only one part of the greater puzzle where many other factors intertwine.^16^ However, we do believe that military physicians play a pre-eminent role in the multidisciplinary teams in screening for serious pathology, providing referrals to other healthcare providers, and in educating recruits in case of MSIs during training.^22 23^ Providing evidence-based, personalised and military-specific care in case of MSIs in recruits is of major importance as recruits must keep up with the military training programme unconditionally.

We should also mention some uncertain factors in this study. We used data that were previously documented by many different military physicians, as part of regular care. Therefore, we had no influence on how data were recorded prospectively. Second, despite the large sample size of both elite military units and the substantial incidence rates, CIs of MSI incidence rates are still relatively wide, reflecting the uncertainty around the point estimations. Third, the frequency of applied primary care interventions could be underestimated due to lack of registration, for example, we assume that provision of injury-specific information and (self-)management options was provided in many more cases, but not explicitly registered. Also, the calculated number of restricted training days per injury is an estimation as nearly one-fifth of all injury-specific activity restrictions were imposed by the military physician using general terms as ‘several days/weeks/months’. In order to calculate restricted training days per injury more precisely in future studies, military physicians should preferably register the exact duration training restriction imposed.

The guidelines of the Dutch College of General Practitioners for knee, ankle and back injuries are well-defined and applicable also in military healthcare; military doctors can individualise the guidelines according to military function and job demands.^10^ However, a National guideline for leg injuries is currently missing. To achieve uniform healthcare for these injuries, the development of a guideline, preferably including a supplementary file for the military population to account for differences in context, would be of value.^24^ Guidelines for treatment protocols and registration of consultations will increase quality, transparency and responsiveness.^25^ In order to develop military primary healthcare standards for MSIs, future research and innovation should focus on prospectively monitoring MSIs and MSI-care in military trainees, development of multifactorial prediction models to recognise a high chance of dropout from training in an early stage, and MSI preventive and primary care intervention studies.

## Conclusion

Our study findings showed that one out of four recruits who dropout from elite military training in the NAF do so due to MSIs. Redesigning training programmes with the objective to reduce MSIs should be given high priority, as this may reduce dropout substantially.

## Data Availability

No data are available.
